# Typification and differentiation of smallholder dairy production systems in smallholder mixed farming in the highlands of southern Ethiopia

**DOI:** 10.1371/journal.pone.0307685

**Published:** 2024-08-29

**Authors:** Tsedey Azeze, Mitiku Eshetu, Zelalem Yilma, Tesfemariam Berhe

**Affiliations:** 1 Hawassa Agricultural Research Center, Hawassa, Ethiopia; 2 Haramaya University, Diredawa, Ethiopia; 3 SNV International Development Organization, Addis Ababa, Ethiopia; 4 Bio and Emerging Technology Institute, Addis Ababa, Ethiopia; Shandong University of Technology, CHINA

## Abstract

The present study aimed to classify smallholder dairy production systems by analyzing land allocation for various crop types and characterizing them based on integration with specific crops which is gap in earlier studies. A total of 360 smallholder farmers were interviewed to generate firsthand information in addition to using secondary data sources. The classification utilized K-means clustering, while discriminant analysis was applied to accentuate the distinct characteristics of dairy production systems (DPSs). Subsequently, for a particular crop to be considered dominant, the minimum farmland allocation had to exceed 30%. Based on this, the 360 respondent farmers in the study areas were categorized into four distinct DPSs: diversified crop-based (DCB) at 31%, enset-based (EB) at 28%, cereal-based (CB) at 24% and cash crop-based (CCB) with coffee, khat, vegetables, fruits, and sugarcane accounting for 17%. Within the CCB dairy production system, where cash crops were cultivated most dominantly, averaging 56% of the farmland, farmers reported the highest annual income and, consequently, acquired more improved dairy cows, facilitating their entry into intensive dairy production. Ownership of improved dairy cattle breeds, use of agro-industrial byproducts as feed, practicing stall feeding and better housing are observed practices in this DPS that relate to the intensification of dairy production. This positive relationship was observed between the commercialization of crops and the intensification of dairy production in the CCB dairy production system. In the EB dairy system, enset dominates with a 33% area share, leading to increased residues for cattle feed. They keep large herds of local cattle, limited crossbreeding experience, and prefer conventional feeding. The CB system allocates 47% of land to cereals, justifying cereal residues as primary dairy feed. Cultivating grasses like Pennisetum pedicellatum and Phalaris arundinacea is also highly valued in the system. The DCB system, with diverse crops (none exceeding 30%), implies varied residues for dairy feed. These findings reveal unique characteristics across dairy systems, indicating a positive correlation between crop commercialization and dairy intensification. Hence, understanding each type of DPS and its characteristics will help development experts or those in charge of designing agricultural policy to establish the best strategic plans for enhancing the productivity of dairy cattle under smallholder conditions.

## 1. Introduction

Ethiopia is Africa’s first and world’s fifth ranked country in livestock population, which in 2017 contributed to about 40% of the agricultural gross domestic product (GDP), and 20% of the national foreign exchange earnings [1]. In 2021/22, Ethiopia’s cattle population reached approximately 66 million heads, with 96.8% owned by smallholder farmers [2]. These smallholder farmers, who typically cultivate less than a hectare of land primarily for subsistence, are also responsible for 95% of the country’s agricultural output, with a focus on cereal crops. This narrative underscores the critical role of smallholders as the main drivers of Ethiopia’s agricultural sector. Additionally, Ethiopia’ produces about 7.13 billion liters of milk annually from cows and camels, with around 65.8% (4.69 billion liters) coming from approximately 7.56 million dairy cows, each average of 1.48 liters of milk per day based on data from November 2019 to November 2020 [2]. The productivity of the dairy cattle in terms of milk yield is generally low in Ethiopia especially under smallholder management condition mainly due to the poor genetic potential of the indigenous cattle breeds for productive traits along with the poor feeding, health care and management practices [3, 4].

Southern Nations and Nationalities Peoples (SNNP) region of Ethiopia is the second largest milk producing region following Oromia region with an estimated total annual yield of about 707.7 million liters registered for year 2021/22 [2]. The recently formed Sidama region is also among the highest dairy potential regions, with an annual milk production of about 268 million liters reported in the same reference year. Over the past few decades, the Ethiopian government, along with various local and international development partners, has initiated, funded, and executed numerous projects related to dairy cattle development. Some of these projects focused on specific geographic areas, while others had regional or even national scopes. Nationwide research by various scholars in Ethiopia has extensively explored the characterization and classification of dairy production, taking into account diverse criteria such as agro-ecology, location, and the integration of crops with livestock production [5, 6]. Ethiopia’s dairy production is categorized into three primary systems: pastoral, agro-pastoral, and sedentary, with classification based on the land use system [7]. Within the sedentary dairy production system, classification factors include location, agro-ecology, primary production objectives, resources and resource utilization, production scale and management, market orientation and access to inputs and services. These criteria are consistently applied across broader dairy production systems, encompassing 1) commercial; 2) peri-urban and urban; 3) rural smallholder mixed crop-livestock; and 4) pastoral and agro-pastoral [5, 6]. Research has highlighted the integration of crop cultivation with livestock production, particularly in the mixed crop-livestock system, agro-pastoral system, and sedentary production system. This integration significantly increases the productivity and income of farming households by up to 50% compared to crop cultivation alone [8]. Additionally, it has been reported to enhance returns on investment for small plots of land and minimize production risks [9]. However, while the importance of integrating livestock with crops is well-recognized, the impact of different types of crop cultivation on livestock productivity, especially dairy production, has not yet been studied in detail. Most research tends to focus on broad categorizations and characterizations without examining how specific types of crop cultivation affect overall livestock productivity. This is a gap that the current study aims to address in our specific study area. Specifically, there is a lack of research demonstrating how variations in crop types and their integration within dairy production influence dairy productivity in Ethiopia, particularly in the highlands of southern Ethiopia. Understanding these relationships is crucial, as the interaction between different crop types and dairy systems can significantly impact dairy production outcomes. This study seeks to address this gap and provide specific recommendations to enhance the productivity of dairy cattle through improved integration of crop types within dairy production systems.

Different types of available crop residues, which are the main source of animal feed, are likely to have different effects on milk production levels as well as milk quality. Assessing the relationship between major crops commonly grown in specific areas and cattle productivity levels within a given production system is essential. By evaluating both the type of crops and the resulting crop residues, we can understand the potential of available feed resources and improve their utilization. This, in turn, can lead to enhanced productivity, improved nutrition, and increased income from dairying in Ethiopia. Furthermore, the utilization of crop residues is a key aspect of dairy production in Ethiopia. As reported by Mengistu et al. [6], residues of various food crops such as cereals, pulses (including faba bean, chickpea, haricot bean, field pea, and lentil), as well as oil crops and by-products of vegetables are commonly used in mid and high-altitude areas, contributing to enhanced productivity. Additionally, studies by Yilma [10] and Senbeta et al. [11] highlight the importance of domesticated enset (Ensete ventricosum, commonly known as false banana) as a significant livestock feed source and staple food crop for millions in southern and southwestern Ethiopia. These findings suggest that understanding the role of major crops cultivated within smallholder farming systems can aid in classifying dairy production systems.

Thus, the present study aims to classify dairy production systems based on the type of major crop cultivated and characterize the classified dairy production systems accordingly. Through these accomplishments, this study contributes to a deeper understanding of the interconnections between crop types and dairy productivity in Ethiopia. Consequently, it offers valuable information that has the potential to enhance dairy production practices

## 2. Material and methods

### 2.1. Study area

The study was conducted in the Sidama and the former Southern Nations, Nationalities and People’s (SNNP) Regional Sates of Ethiopia The Sidama is located between 5°45’ to 6°45′N latitude and longitude of 38°5’- 39°5′E while SNNP regional state is located between 4°26′ to 8°27′N latitude and longitude of 35°45′-38°42′E. Both regions encompass two major agroecological zones: midland and highland. The midland zone has an altitude ranging from 1500 to 2300 meters while the highland zone is situated at elevations between 2300 to 3200 meters [12].

The rainfall in both regions is bimodal where the short rainy season occurs from February to May while the main rainy season occurring from June to September [13–15]. The average annual rainfall in the study areas of Sidama ranges from 801 to 1600 mm while 400mm to 2200mm in SNNP region [16]. The major food crops in both regions are enset (*Ensete ventricosum*) from perennial crops and potato from annual crops while khat (*Catha edulis*) whose leaves are consumed for their stimulant effect, vegetables (cabbage) and coffee (*Coffee arabica*) are the major cash crops [17].

### 2.2. Selection of study sites and farm households

For the current study, two regions, namely SNNP and Sidama from the southern part of Ethiopia, were purposefully selected due to their potential in dairy production. The study’s significance in dairy production lies in the purposeful selection of these regions. These regions are known for their significant contribution to the country’s dairy sector, characterized by favorable agro-climatic conditions and suitable land for grazing. Although formal zonal level administrative clustering is absent for the Sidama region, five proposed zones were identified. Meanwhile, the SNNP region comprises 17 zones. Consequently, one zone from Sidama and two zones from SNNP were also purposefully selected, based on their potential dairy cattle production. Following this zonal selection, six districts were chosen for the study, with four selected from the SNNP region and two from Sidama. These selections were made purposefully, considering the dairy production potential at both the zonal and district levels. Employing a systematic random sampling approach, a total of 12 kebeles (Lower administrative unit), with two selected from each district, were chosen. The sampling interval was determined based on the geographic distance between kebeles, ensuring comprehensive representation across the district. The identification of kebeles involved in selecting those located both in near to and farthest from a central marketplace, which served as a strategic reference point within the districts. To achieve this, distances from the major marketplaces in each district, as well as the altitudes of each household, were measured using the Global Positioning System (GPS) along roads and footpaths. In the context of this study, ’distance’ is defined as the measurement from the major livestock feed markets, located in the towns of each district, to both the nearest and farthest potential kebeles for dairy cattle within the district. The distances of the kebeles along market gradients were determined based on traveling time using vehicles such as Bajaje (a three-wheeled scooter) and motorcycles. The consideration of major marketplaces in the study areas is associated with the availability and accessibility of animal feeds particularly agro-industrial by-products as well as market outlet for producer households’ surplus milk and milk products. The major marketplace for the target kebeles from the Wondo Genet district is located in the Basha town and for those from Melga district is located in the Guguma town while for Damot Gale and Soddo zuria districts from Wolita zone, Boditi and Lasho towns serves as the major marketplaces, respectively for the selected target kebeles (Table 1). For the selected kebeles of Kedida Gamela District, Durame town is taken as a major marketplace.

**Table 1:** Agro-ecology, elevation, and distance to major marketplaces of target sample kebelles by district.

Districts	Kebele’s	Major market place		Agro- ecology	Elevation (av.) (meter above sea level)	Distance to market (km)
Kedida Gamela	Zato Shodora		Durame town	Midland	2030	Nearby
	Dega Kedida			Midland	2156	Distant
Domboya	Kezala		Domboya town	Highland	2343	Nearby
	Heba			Highland	2376	Distant
Damot Gale	Shasha Gale		Boditi town	Midland	2114	Nearby
	Wendara Boloso			Midland	2056	Distant
Soddo zuria	Lasho		Lasho town	Highland	2349	Nearby
	Gurumo Weyede			Highland	2403	Distant
Melga	Guguma		Guguma town	Highland	2671	Nearby
	Wotera			Highland	2669	Distant
Wondo Genet	Wesha		Basha town	Midland	1746	Nearby
	Abaye			Midland	1834	Distant

Kebele = Lower administrative unit; Elevation = average elevation of sampled households in the kebeles

As a result, recorded distances ranged from 3 to 27 km, with distances under 10 km being regarded "nearby" and ones over 10 km being considered "distant". A systematic random sampling technique was employed to select sample farming households within each kebeles. Farming households who have at least one milking cow were first identified purposefully from the list obtained from the respective district offices. Following that, target sample households were selected from the list using two formulas, the first of which was used to determine the sample size according to Eq 1 [18]. The second equation was to ensure the systematic random sampling interval of the households [19].


$$n=\frac{N}{1+N(e^{2})}$$ Eq 1

$$i=\frac{N}{n}$$ Eq 2

Where N = Population size (3600); n = required sample size, and e = precision level (0.05), i = systematic random sampling formula for household selection interval. Accordingly, a total of 360 target sample households were randomly selected from all target districts and kebeles, and households with cattle were sampled at intervals of every 10 households (i), extending the interval until the target sample size was attained within each district boundary.

### 2.3 Ethics statement

The study was ethically approved by Southern agricultural research Institute (SARI) and prior to the start of the survey, farmers were informed of the study’s purpose, the importance of the information they provided, the confidentiality of their information, and their freedom to decline or withdraw from research. Farmers were reassured that there was no risk in participating in the study, and they were specifically informed about the anonymous handling of individual replies.

### 2.4. Data collection

The data were collected from February to April 2022. The required data for this study were collected from primary and secondary sources. Primary data were collected through household survey, following the pretesting and subsequent modification of the semi-structured questionnaire. Primary data were collected through a household survey, following the pretesting and subsequent modification of a semi-structured questionnaire. In our study, we collected comprehensive data on household characteristics, encompassing family size, age, and education levels. We also gathered information on resource endowment, including farm size, herd size, and the allocation of land to various crops such as annual cereals, root and tuber crops, pulses, enset, khat, coffee, vegetables, and grazing land. Additionally, we examined details related to feed resources, feeding methods, cattle breeds, breeding practices, milk yield and production, and various aspects of cattle management, including health services, housing, and the management of organic waste on dairy farms. To standardize livestock units across different species, we employed Tropical Livestock Unit (TLU) conversion factors: 1 cattle head = 0.7 TLU, 1 sheep/goat = 0.1 TLU, 1 donkey = 0.6 TLU and chicken = 0.01 TLU [20]. A semi-structured questionnaire was prepared in 3 local languages, namely Sidamigna, Wolaitigna, and Kembatigna, and pretested before the actual survey. Published literature also used as the main secondary source of information.

### 2.5 Farm typology

Dairy production typology was determined by considering the share of various crops and grazing areas. Nine crop varieties (*ense*t, coffee, *khat*, sugarcane, vegetables, fruits, cereals, root crops, and pulse) and grazing area share derived from the total land size in the land certificate book were considered in the cluster analysis. In addition, grazing lands and other land occupations (i.e. homestead and woodland) were considered for determining total household land holding. However, as land occupied by homestead and woodland do not have direct relation with animal feeding and it was rejected for the current clustering analysis. The naming of dairy production systems in the current study is based on the type of crop cultivated on at least 30% of the farmland. Any percentage below 30% is classified as a diversified dairy production system, represented by the DCB in the current study, while percentages above 30% are labeled as specific crop-dominated dairy production systems. This approach is consistent with the methodology used by Mellise et al. [21], who utilized similar criteria for classifying home gardens based on crop area share.The same variables were employed to highlight smallholder dairy production system through discriminant analysis.

### 2.6 Data analysis

A clustering analysis using K-means classified the four types of dairy production systems, and ANOVA was employed to analyze differences in various dependent variables, including family size, farm size, herd size, livestock numbers, cattle, annual income, distance from marketplaces, feed sources index values, milk productivity, and annual milk production among the identified systems. The K-means clustering method was used to analyze the different types of farms, taking into account the area proportion of crops grown in 2020/21 and grazing land [22–24].

The data collected on these variables were analyzed using the Statistical Package for the Social Sciences (SPSS) version 25, with significance considered at P<0.05. Additionally, Chi-square values were applied to qualitative parameters such as available feed types, breed type and breeding method, access to dairy cattle health services, and dairy cattle housing type. Moreover, for the feed sources rank where responses were stated in the form of a five-point Likert scale, the results were first standardized to a common scale value before analysis. The Likert scale responses ranged from 1 up to 5. The standardization of the five-point Likert scale responses to a common scale was performed using the method described by Tessema and Simane [25].

## 3. Results

### 3.1 Characteristics of dairy production systems

Although, smallholder dairy production is the predominant system in Ethiopia, intra-diversity was observed in the study area. The four production systems identified within the smallholder dairy production system in the study area are 1) diversified crop based (DCB), 2) cereal based (CB), 3) cash crop based (CCB) and 4) *Enset* based (EB) dairy production systems. Based on the K-means clustering analysis outcomes, DCB system accounted for 31%, EB for 28%, CB 24%, and CCB for 17% within the smallholder DPS in the study areas. In the CCB system, cash crops occupy more than half of the total farm area. The grazing land share in the CCB dairy production system is approximately half that of the DCB and EB dairy production systems, indicating a significant disparity in grazing land availability Smallholder farmers in the EB dairy production system are allocated more than one-third of their farm to *enset* (staple perennial food crop), while in the CB dairy production system, annual Cereals occupy about half of the farm area. Additionally, in the CCB and CB dairy production systems, *enset* is the second most important crop though occupies smaller farm area. Finally, the DCB occupied less than 30% of the total farm land holding (Table 2).

**Table 2:** Characteristics of dairy production systems.

Description	Variables	Types of Dairy production systems (DPS)			
		CCB (N = 60)	EB (N = 101)	CB (N = 87)	DCB (N = 112)
Share of farm area (%)	Enset	17.12±1.70	**33.25±1.15**	13.14±0.67	10.44±0.67
	Cash crops	**55.86±1.84**	10.70±0.99	7.17±0.90	11.38±0.84
	Annual cereals	6.15±1.37	13.31±1.06	**46.68±1.45**	16.77±0.95
	Root & pulse crops	1.37±0.54	9.38 ± 0.89	6.40±0.85	21.01±1.31
	Grazing lands	6.47±1.13	12.31±1.17	8.88±0.95	12.78±1.17
	Other	13.02±1.24	21.04±1.52	17.72±1.37	27.62±1.96
HH Characteristics	Family size (#)	7.23±0.34^a^	7.09±0.28^a^	6.59 ± 0.28^b^	6.06±0.24^b^
	Farm size (ha)	1.46±0.23^ab^	1.66±0.16^a^	1.11 ± 0.09^b^	1.35±0.13^a^
	Livestock (TLU)	3.32±0.29	3.74±0.29	3.02 ± 0.22	3.27±0.27
	Cattle (TLU)	3.26±0.28	3.32±0.27	2.70 ± 0.21	2.92±0.24
	Improved breed (TLU)	2.02±0.29^a^	1.03±0.14^c^	1.46 ± 0.18^b^	1.09±0.11^bc^
	Local breeds (TLU)	1.24±0.21^b^	2.29±0.27^a^	1.24 ± 0.16^b^	1.83±0.2^ab^
	Annual income (US$)	3982.9±552.5^a^	1803±190.5^b^	1994.5±271.3^b^	1809.3±186^b^
	Distance from market (km)	5.03±0.44^b^	9.08±0.63^a^	9.53±0.80^a^	10.58±0.70^a^

CCB = Cash crop based; EB = Enset based; CB = Cereal based; DCB = Diversified crop based; TLU (Tropical Livestock Unit); Different letters in a column indicate significant differences; No superscript for household characteristics means no significance difference; Cash crops include coffee, khat, vegetables, fruits, sugarcane, others include homestead, and woodland; TLU = Tropical Livestock Unit; Average exchange rate for 2021: 1 USD = 44 Ethiopian birr.

Farmers in the CCB and EB had significantly higher family size compared with that in the CB and DCB dairy production systems. Although there was no variation in total livestock and cattle population, difference were observed within breed type (improved and local breed) across the dairy production systems. Accordingly, the farmers in the CCB dairy production system had significantly more improved dairy cattle compared with that in the other three dairy production systems. Sample farmers in EB system, on the other hand, had significantly more local cattle breeds compared with that in the CB and CCB dairy production systems. Moreover, household annual income was significantly higher for the CCB compared with that in the other three dairy production systems. Farmers in the CCB dairy production systems were more proximate to major marketplaces compared with those in the three DPSs.

**Fig 1:**
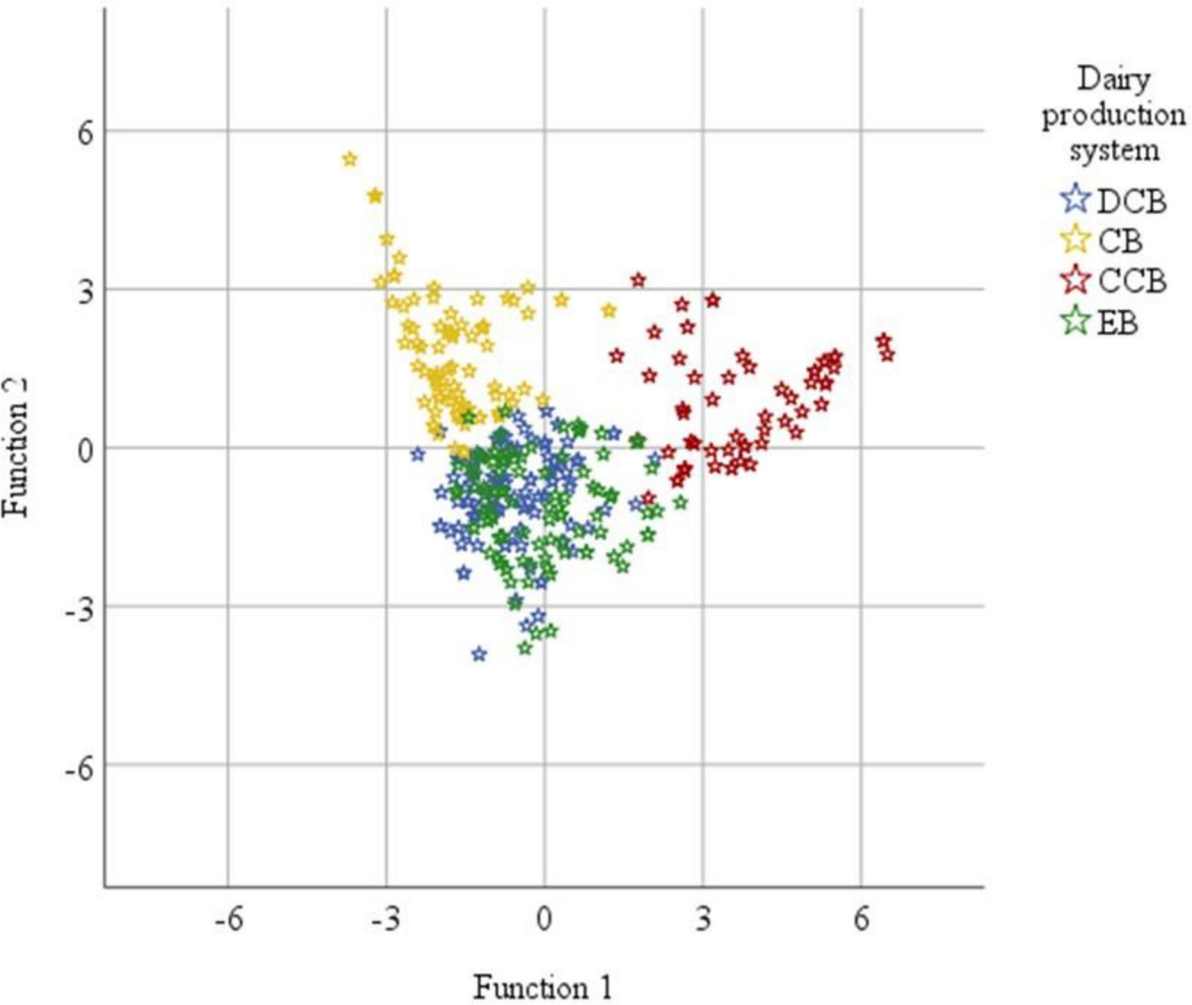
Canonical correspondence analysis map of dairy production systems.

Additionally, the discriminant analysis, considering the area allocated to various crops, revealed clear distinctions among the four dairy production systems. The Cereal-Based (CB) and Cash Crop-Based (CCB) systems occupy entirely different domains, potentially attributed to the minimal share of cash crops in CB and the lowest annual cereal farm shares in CCB (Table 2; Fig 1). However, the Enset-Based (EB) and Diversified Crop-Based (DCB) systems exhibited overlap, positioning them in separate spheres compared to the CB and CCB dairy production systems.

### 3.2 Distribution of the dairy production systems across agro ecology and study districts

**Fig 2:**
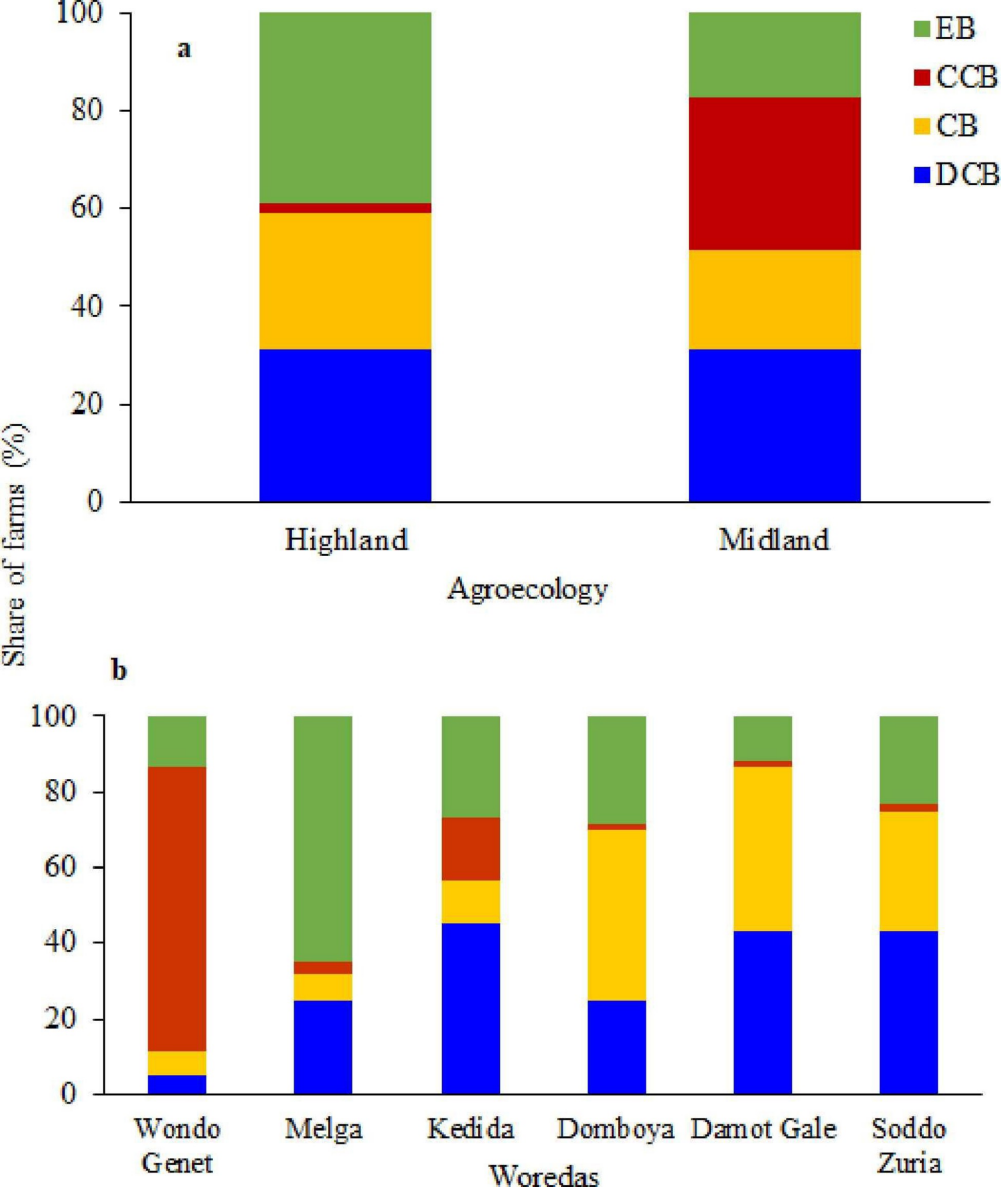
Distribution of Dairy production system types in agro ecology (A) and study Districts (B).

All the four dairy production systems were almost equally distributed in both midland and highland agro ecologies, with the exception of the cash crop based dairy production system, which is less common in the highland agro ecology (Fig 2A).

The distribution of the four dairy production systems varied across the six districts (Fig 2B). Wondo Genet district was dominated by CCB dairy production system while EB was the most dominant dairy production in the Melga district. The two dairy production systems such as CB and DCB were fairly distributed in Damot Gale while DCB was dominated in Kedida district. Districts of Domboya and Sodo Zuria are more represented by all dairy production system except CCB. In Wondo Genet, farmers are predominantly commercial, mainly engaging in CCB dairy production. Conversely, in the Melga district, which is primarily occupied by enset-cultivating farmers, the prevalent dairy production system was EB.

### 3.3. Feed resource and feeding method

#### 3.3.1 Feed resources

In the study areas, the major dairy cattle feed resources identified included natural pasture, crop residues, improved forage crops, agro-industrial byproducts, and non-conventional feed resources. These resources exhibited varying patterns of utilization among the four dairy production systems, as outlined in Table 3. The term ’non-conventional feed resources,’ specifically refers to brewery residues, homemade alcoholic drink residues, and household wastes, as illustrated in Table 4. In EB production system, farmers primarily rely on natural pasture compared to the other three dairy production systems. Conversely, there was no significant difference observed in the utilization of crop residues. The dependency on improved forage varies among dairy production systems, with farmers in CB system showing a notably higher reliance compared to CCB and EB systems. Furthermore, farmers in the CCB system demonstrate a higher utilization of agro-industrial by-products such as concentrates, wheat bran and Nouge cake, compared to those in the EB system. Conversely, farmers in the EB significantly rely on non-conventional feed sources such as brewery spent grain, local beverage by-products and household food wastes, in comparison to those in the CCB dairy production system.

**Table 3:** Index value for major feed resources for dairy cattle (mean±SE).

Feed resources	Dairy Production system			
	CCB (N = 60)	EB (N = 101)	CB (N = 87)	DCB (N = 112)
Natural Pasture	0.79 ± 0.025^b^	0.86 ± 0.017^a^	0.79 ± 0.019^b^	0.77 ± 0.017^b^
Crop Residue	0.66 ± 0.025	0.71 ± 0.018	0.69 ± 0.019	0.71 ± 0.015
Improved Forages	0.42 ± 0.036^c^	0.54 ± 0.027^b^	0.61 ± 0.024^a^	0.57 ± 0.023^ab^
Agro-industrials byproducts	0.54 ± 0.032^a^	0.40 ± 0.033^b^	0.47 ± 0.031^ab^	0.49 ± 0.030^a^
Non-Conventional	0.26 ± 0.024^b^	0.39 ± 0.017^a^	0.32 ± 0.027^ab^	0.28 ± 0.035^ab^

Values with different letter across the column indicted significant difference at p<0.05, values with no superscripts indicates that there is no significant variation CCB = cash crop based, EP = Enset -based CP = Cereal based, and DCP = Diversified crop based

**Table 4:** Dairy cattle feed types available in each dairy production systems.

Feed resources	Feed types	Dairy Production Systems							
		CCB (N = 60)		EB (N = 101)		CB (N = 87)		DCB (N = 112)	
		F	%	F	%	F	%	F	%
Natural Pasture	Legume herb dominated	60	15.4	101	25.9	87	22.3	112	28.7
	Herb dominated	5	1.3	11	2.8	2	0.5	12	3.1
	**Total**	65	16.7	112	28.7	89	22.8	124	31.8
Improved forage	Elephant grass (*Pennisetum purpureum*)	16	6.1	15	5.7	21	7.8	28	10.6
	Desho *(Pennisetum pedicellatum*)	23	8.7	54	20.5	78	18.4	57	13.5
	Phalaris *(Phalaris arundinacea)*	7	2.7	15	5.7	34	12.6	17	6.4
	Alfa alfa *(Medicago sativa)*	4	1.5	8	3.0	4	1.5	4	1.5
	Guatimala *(Tripsacum andersonii)*	5	1.9	2	0.8	2	0.8	2	0.8
	Sesbania *(Sesbania Sesban*)	0	0	2	0.8	0	0	5	1.9
	Rhodes *(Chloris gayana)*	0	0	0	0	5	1.9	5	1.9
	Panicum *(Panicum maximum*)	0	0	0	0	3	1.1	0	0
	Desmodium *(Desmodium intortum)*	1	0.4	1	0.4	1	1.4	2	0.8
	**Total**	56	13.2	97	22.9	148	35.0	122	28.8
Crop residue (Cereal)	Wheat	6	0.9	40	5.9	75	11.0	67	10.1
	Barely	3	0.4	50	7.3	25	3.7	21	3.2
	Sorghum	0	0	2	0.3	11	1.6	3	0.5
	Teff	27	4.0	37	5.4	66	9.7	57	8.6
	Maize	46	6.8	48	7.0	57	8.4	40	6.0
	**Total**	82	12.0	177	26.0	234	34.4	188	28.2
Crop residue (Pulse)	Haricot bean	7	4.9	5	3.5	26	18.2	32	22.4
	Faba bean	0	0	19	13.3	26	18.2	16	11.2
	Field pea	0	0	2	1.4	6	4.2	4	2.8
	**Total**	7	4.9	26	18.2	58	40.8	52	36.4
Crop residue (Root crops)	sweet potato	0	0	7	1.9	10	2.7	22	6.0
	*Enset*	55	15.1	99	27.2	70	19.2	75	20.6
	Cassava	3	0.8	3	0.8	1	0.3	2	0.5
	Taro	0	0	2	0.5	6	1.6	9	2.5
	**Total**	58	15.9	111	30.5	87	23.9	108	29.7
Crop residue (others)	Sugarcane top	33	22.4	15	10.2	11	7.5	17	11.6
	Banana leaf	16	10.9	13	8.8	8	5.4	16	10.9
	Avocado leaf	1	0.7	4	2.7	5	3.4	8	5.4
	**Total**	50	34.0	32	21.8	24	16.3	41	27.9
Non-conventional	Brewery residue drinks	7	3.5	5	2.5	2	1	0	0
	Homemade alcoholic residue (Atella)	2	1.0	6	3.0	9	4.5	2	1.0
	House wastes	8	4.0	44	22.0	47	23.5	68	34.0
	**Total**	17	8.5	55	27.5	58	29.0	70	35.0

F = Frequency; Percent values are calculated separately for each feed resources, with the total summing to 100% across all four dairy production system.

In addition to the major available feed resources, several feed types within feed resources were identified under each production systems (Table 4). The natural pasture as one of the major feed resource, the legume herb types such as *Trifolium ruppellianum*, *Dicrocephala integrifolia*, *Bidens macroptera* were the dominantly (>90%) used in the four dairy production systems. Regarding improved forage type resource, the highest proportion of sample respondents in the three DPS reported to mainly use desho grass though the degree varies between the systems with its plantation and utilization being the least among farmers in the CCB system. The next commonly cultivated and utilized improved forage types were elephant and phalaris grasses in all dairy production systems. Smallholders in the CB system also have better access to improved forage crops for their dairy cattle than smallholders in the other DPSs. Sesbania (*Sesbania Sesban*), Rhodes (*Chloris gayana*), Panicum (*Panicum maximum*), and Desmodium (*Desmodium intortum*) were some of improved forage varieties that were less frequently cultivated and utilized in all of the dairy production systems in the study.

The use of crop residue is based on common crops cultivated in the study areas’ dairy production systems. Thus, cereal crop residues were more readily available in the CB while receiving less reporting in the CCB dairy production system. Despite the fact that overall root crop residue and one of their components, *enset* crop residue, are both more readily available in the EB, root crops are often more readily available in the DCB than the other DPSs. The CB and DCB contain the most wheat straw, whereas the EB dairy production systems contains the most barley straw. However it seems like every dairy production system has easy access to maize crop residues.

Furthermore, sugarcane (one of the cash crops in the current study), its top part is mainly used in the CCB relative to the other DPSs. Brewery spent grain, local beverage residues and kitchen organic wastes were also reported as non-conventional feeds common in the current study areas. The brewery spent grain was dominantly utilized as dairy cattle feed especially for lactating cows in CCB system.

#### 3.3.2 Feeding methods

The three feeding systems practiced by the farmers in the four dairy production systems were free grazing, stall-feeding, and tethering for both type of breed (Table 5). Free grazing was significantly less practiced by farmers in CCB system for both types of breeds compared to the other three dairy production systems, while stall feeding was significantly more practiced. The dependency of smallholder farmers on natural pasture grazing was higher in EB for both local and cross breeds followed by tethering. In contrary, more farmers in DCB practice tethering for cross breeds compared to farmers in the other three dairy production systems.

**Table 5:** Feeding methods for local and crossbreds in the study areas (frequency/ %).

Breed	Feeding methods	Dairy Production systems								χ2value/ P-Value
		CCB		EB		CB		DCB		
		F	%	F	%	F	%	F	%	
Local	Grazing	22	11	71	34	58	28	59	28	33.317/0.00
	Stall-feeding	23	32	14	19	16	22	19	26	30. 664/0.00
	Tethering	28	13	68	32	54	26	62	29	5.175/0.417
Crossbred	Grazing	8	6.3	42	33	38	30	38	30	48.540/0.00
	Stall-feeding	48	29	33	20	41	25	43	26	22.848/0.00
	Tethering	31	16	44	23	56	29	62	32	17.489/0.001

### 3.4 Breed and breeding practices

Farmers in the four dairy production systems practiced natural mating, cross breeding, and a combination of both (Table 6). Natural mating (uncontrolled natural mating) was more practiced in EB as compared to the other three production systems while crossbreeding was more practiced among farmers in the CB followed by farmers in the CCB dairy production system. More than half of the respondent farmers in the DCB and CB system use artificial insemination (AI) solely for crossbreeding, whereas about 85% of the farmers in the CCB used both AI and natural mating with an improved bull, as well as AI exclusively. Although there was no clear variation in the proportion of respondents that own crossbred cattle among the four dairy production systems, more farmers (67%) reportedly own crossbreds in the CCB compared with the other three dairy production systems. In terms of the specific crossbreeds owned, more than three-fourths of the farmers in the CCB had local crossbreeds between local breeds and Holstein-Friesian (HF), whereas it was less than half for those in the other three dairy production systems. Additionally, crossbreeding practices also involved local breeds being crossed with Jersey, as well as combinations of both Holstein-Friesian and Jersey with local breeds. Moreover, Jersey crossbreeds were more common among farmers in the DCB and CB dairy production systems compared to those in the CCB and EB dairy production systems.

**Table 6:** Breed types owned and breeding methods used by smallholder farmers.

Description	Types of DPSs								Total		χ2/P-value
	CCB		EB		CB		DCB				
	N = 60		N = 101		N = 87		N = 112		N = 360		
	F	%	F	%	F	%	F	%	F	%	
Breeding method used											
*Natural mating*	20	33	49	49	32	37	43	38	144	40	6.486/0.37
*Crossbreeding*	24	40	34	34	38	44	38	34	134	37	
*Both*	16	27	18	18	17	20	31	28	82	23	
Crossbreeding method used											
*Bull service only*	6	15	12	23	10	20	7	10	35	17	16.80/0.01
*AI only*	18	45	21	40	36	69	36	52	111	52	
*Both*	16	40	19	36	6	12	26	38	67	31	
Farmers with crossbreds	40	67	52	52	52	60	69	62	213	59	4.15/0.25
Breed type owned											
HF*Local	31	78	24	46	21	40	27	39	103	48	26.09/0.00
Jersey*Local	4	10	18	35	25	48	37	54	84	39	
*Both*	5	13	10	20	6	12	5	7.3	26	12	

HF (Holistein Friesian)

#### 3.4.1 Crossbred dairy cattle ownership and milk production

Farmers in the CCB DPS had significantly larger number of crossbred cows during the past five years (2017–2021 GC) compared with those in the other three dairy production systems. No significant difference was observed in milk yield of local cows among the four dairy production systems. Crossbred cows in the CCB, however, exhibited significantly higher daily milk yields compared to those in the DCB system during the wet season (Table 7). This difference in daily milk yield suggests notable variation among the dairy production systems, emphasizing the superior performance of the CCB system.

**Table 7:** Dairy cattle ownership for the last five year (2017-2021GC) and milk production across dairy production system (Mean±SE).

Description	Types of DPS			
	CCB (N = 60)	EB (N = 101)	CB (N = 87)	DCB (N = 112)
Crossbred cattle ownership (#)	2.85±0.43^a^	1.22 ±0.16^b^	1.83±0.22^b^	1.54± 0.17^b^
*Daily milk yield of local cows (liters/cow/day)*				
Dry Season (December to February)	1.04±0.09	0.98±0.08	1.04±0.08	1.12±0.08
Wet Season (All months of a year except Dry)	1.94±0.16	1.88±0.10	1.88±0.12	2.13±0.11
Average	1.49±0.11	1.45±0.08	1.49±0.09	1.63±0.09
*Daily Milk yield of crossbreds (liters/day/cow)*				
Dry Season	6.11±0.46	5.24±0.44	5.53±0.42	5.07± 0.45
Wet Season	11.24±0.87^a^	9.60±0.56^ab^	9.62±0.54^ab^	9.03±0.44^b^
Average	8.57±0.63^a^	7.42±0.47^ab^	7.58±0.47^ab^	7.04±0.43^b^

Values with different letter across the row indicted significant difference at p<0.05; values with no letter across the row indicates that there is no significance difference

### 3.5. Access to dairy cattle healthcare services

Dairy cattle health service is mainly provided by the public sector with less than 20% of the sample respondent farmers reported to also get the service from both public and private service providers except in the EB system where about 31% get service from both service providers (Table 8). More than 80% of interviewed farmers in CCB, CB and DCB received health services mainly from public providers, unlike the EB system where private service provider also operate. Animal health care practitioners sometimes provide on-site services at farm gates. However, in over 50% of the cases, farmers must bring their animals to the nearest veterinary health service center. In the CCB system, these centers are often located less than half a kilometer from the farmers’ gates, contrasting with the other systems. Additionally, in the CCB system, animal health care providers visit farming households more frequently, this differs from the practice in other dairy production systems.

**Table 8:** Veterinary health services across dairy production system.

Description	Dairy production systems								χ2/P-value
	CCB		EB		CB		DCB		
	F	%	F	%	F	%	F	%	
*Service providing institutions*									
*Public*	53	88.3	70	69.3	73	83.9	90	80.4	10.378/0.02
*Private and public*	7	11.7	31	30.7	14	16.1	22	19.6	
*Distance of VHS providers*									
< 50 meter	5	8.3	5	4.9	4	4.6	2	1.8	14.253/0.11
100 meter	6	10	11	10.9	2	2.3	8	7.1	
100–500 meter	18	30	29	28.7	30	34.5	25	22.3	
>500 meter	31	51.7	56	55.5	51	58.6	77	68.8	
*VHS getting method*								
By taking the cattle to VHS center	17	23	87	57.2	72	59.5	96	57.1	87.27/0.00
The service provider comes to the farm	57	77	65	42.8	49	40.5	72	42.9	26.62/0.00

χ2 = chi-square value

### 3.6 Dairy cattle housing and waste management

#### 3.6.1 Dairy cattle housing

In the present study, two types of housing for dairy cattle were identified to be used by respondents across the dairy production systems. These are animals, sharing the same house with the family and 2) a separate house for cattle. Accordingly, 90%of the respondents in the CCB reportedly own a separate house for their cattle whereas the lowest proportion, 45% was reported by the sample respondents in the CB dairy production system. More than 80% of the farmers in the DCB and CB DPSs reported to use bare soil floor while 65% of the farmers in CCB use concrete base floor. About half of the farmers in EP use either earthen floor or wooden made floor. More than 70% of the farmers in DCB, CB and CCB DPSs use wood to construct the wall and iron sheet for the roof while about half of the farmers in EB DPS use wood to construct the wall and iron sheet and the reaming half use wood to construct both the wall and the roof. Straw or grass was the major bedding materials for the farmers in DCB and CB DPSs while straw or grass and concrete for those in CCB DPS and wooden shaving for those in EB Dairy production system.

#### 3.6.2 Manure management

**Fig 3:**
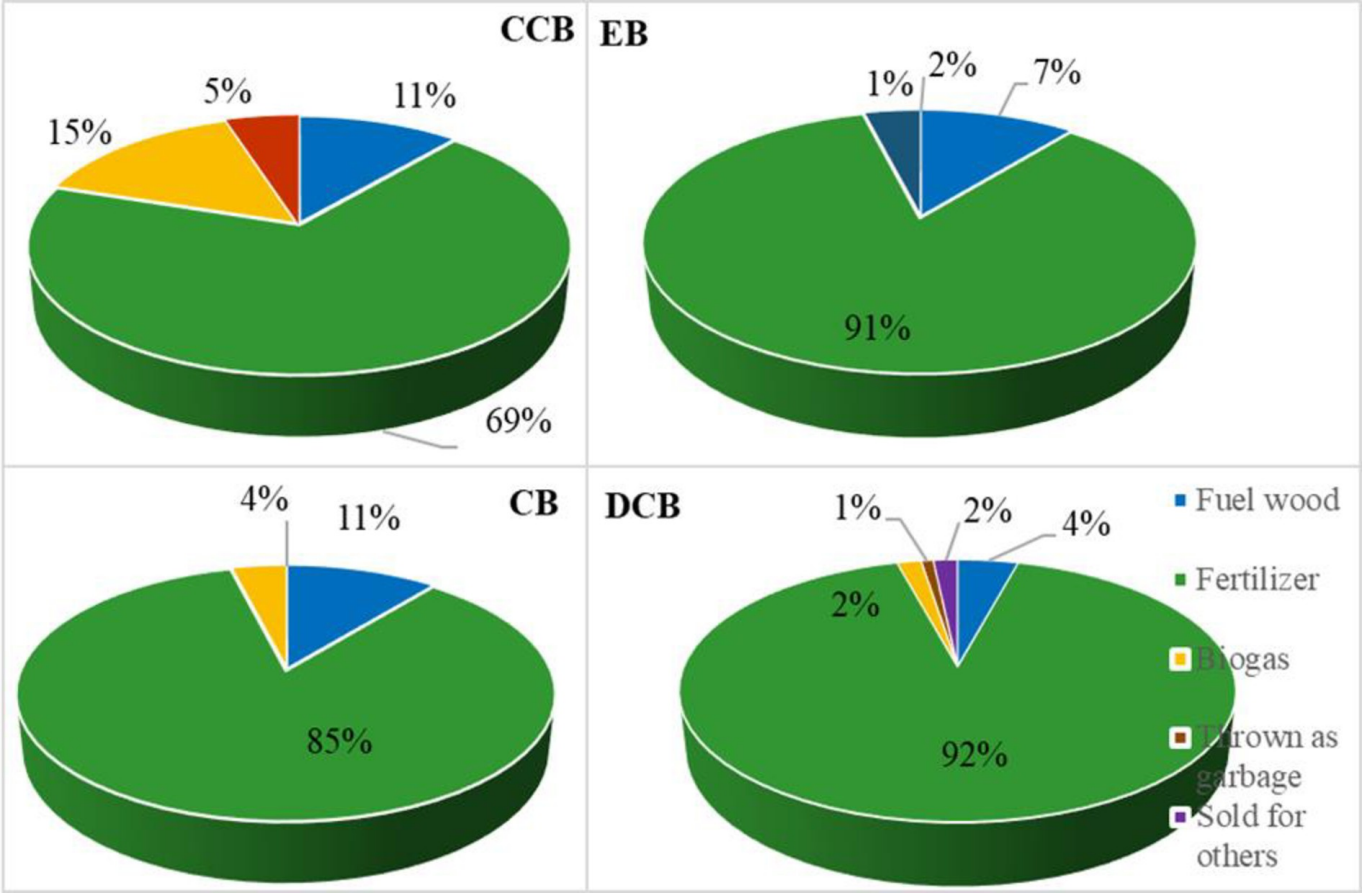
Manure management among smallholder farmers in the study areas.

Smallholder farmers in the study areas apply different cow dung or manure management systems based on the type of the dairy production system (Fig 3). Smallholder farmers who apply cow to crops as a fertilizer were 85% in CB, 92% in DCB, 69% in CCB and 91% in EB DPSs. More farmers in CCB use cow dung as biogas compared to those in other three DPSs.

## 4. Discussion

### 4.1 Diversity in smallholders’dairy production systems

Ethiopia’s smallholder dairy production systems are commonly categorized into mixed, pastoral and agro-pastoral, urban and peri urban and commercial systems [5, 26]. Previous studies have provided more detailed classifications based on agro-ecology, location, and integration with crop farming [7, 27, 28]. For instance, one study broadly categorized dairy production system into Pastoral, agro pastoral and sedentary systems, with the sedentary further subdivided in to urban, peri-urban and rural systems [7]. Another study provided a similar classification of the sedentary category as main dairy production category, based on the agro-ecology and location [27]. These all earlier studies, however, remained in broader categories and did not account for the integration with specific types of crops dominantly grown in different areas, which could have direct or indirect effects on dairy production. Building on these frameworks, the present study explored deeper into the diversity of smallholder dairy production systems by conducting a cluster analysis that considered the dominant crops cultivated in specific areas (Table 2), while the earlier ones only reflecting the prevalent interaction between livestock and crop production in Ethiopia [5, 7, 29, 30]. Smallholders’ tradition of practicing integrated crop-livestock production stems from the important contribution of crop residues, such as *enset*, a perennial staple food in mitigating the recurrent challenge of feed shortage in the study areas [31]. This study identified four distinct dairy production systems, distinguished by the types of crops that are combined with cattle, as shown in Table 2. Differentiating the diversity of smallholder dairy production system based on their integration with crops and identifying the distribution of the farm types across different landscapes paves the way to designing tailored development options.

**Fig 4:**
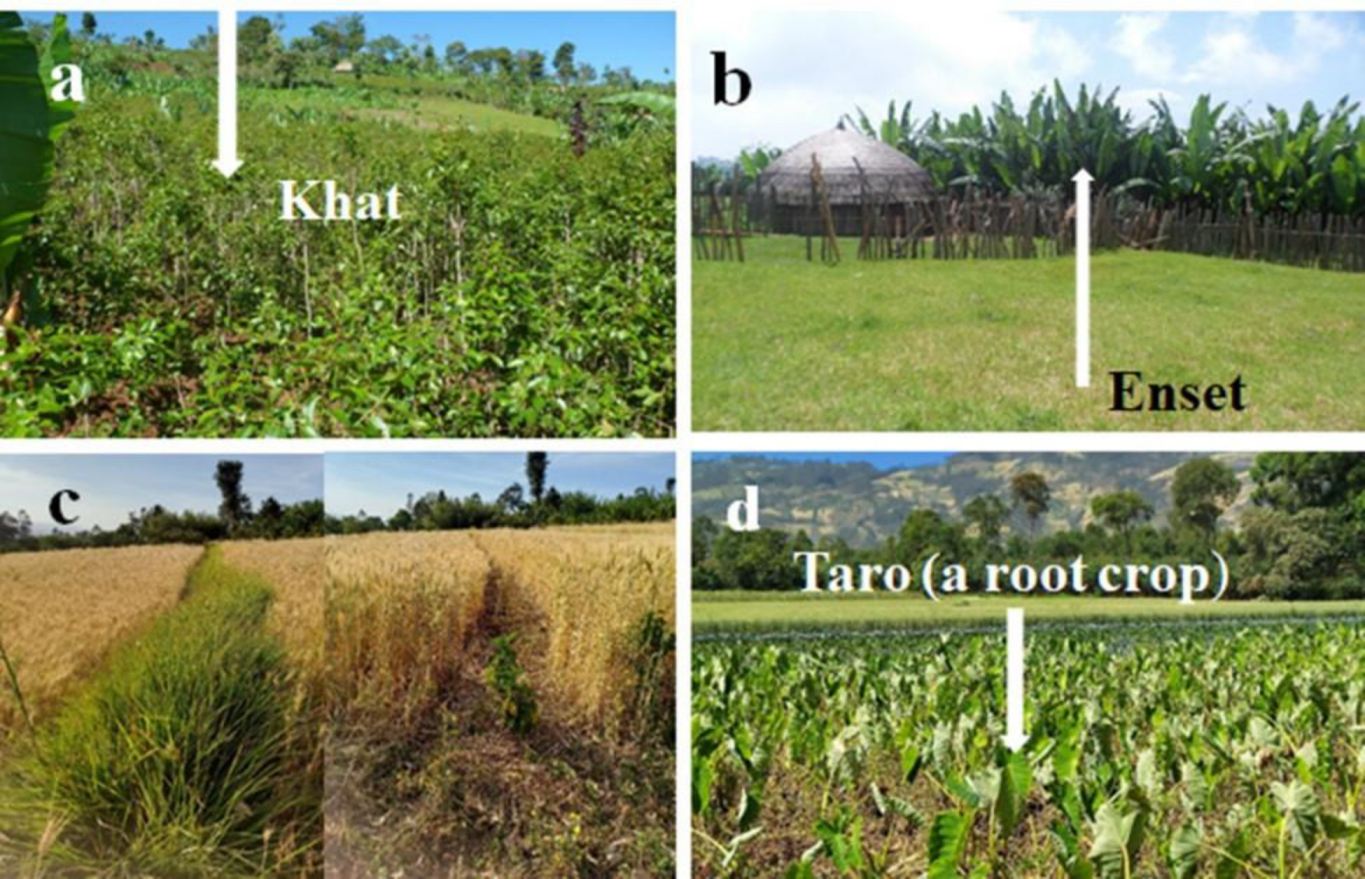
Cash Crop Based (A), Enset Based (B), Cereal Based (C) and Diversified Crop Based (D) DPSs.

Four dairy production systems namely Cash Crop Based (CCB), *Enset* Based (EB), Cereal Based (CB) and Diversified Crop Based (DCB) dairy production systems were identified in the current study areas. Farmers identified under the 3 (CCB, EB and CB) dairy production systems differ in the type of crops that they dominantly allocated in their respective farms (Fig 4). Farmers in CCB dairy production system allocated more than half of their farmland to cultivate cash crops such as *khat* (*Catha edulis*, whose leaves are chewed for their stimulating effect), coffee, sugarcane, fruits and vegetable, while those in EB DPS allocated one-third of their farmland for *enset* (*Ensete ventricosum*), staple food crop in the study areas and those in CB dairy production system allocated about half of their farm to cereal crops (Fig 4, Table 2). In the DCB dairy production system, the proportion of land allocated to crops is below 30%, falling short of the threshold considered for naming a Dairy Production System (DPS). Previous research has classified smallholder dairy production systems in developing nations into categories such as smallholder systems, smallholder cooperative dairy production systems, and intensive systems [9]. Similarly, a study in Kenya differentiated between intensive and semi-intensive dairy production systems [32]. While both studies primarily characterized systems based on feed and feeding methods, which, despite not being the primary classification criteria in the present study, is still regarded as one of the parameters for system characterization. Moreover, a study conducted in the Brazilian semiarid region categorized smallholder dairy production systems into three categories: conventional, traditional, and emerging. While the classification criteria themselves differed from those of our study (Table 2) the fundamental variables utilized for categorization, such as herd size, milk production, and aspects like technology utilization and management methods, closely resembled those employed in our research [33]. Thus, while the present study’s classification of smallholder dairy production systems align with the characterization criteria mentioned in earlier studies to describe the systems, it’s noteworthy that our classification methodology uniquely centers on the types of crops cultivated. This emphasis sets our study apart from previous Ethiopian research, including studies by Azage, Gizaw, FAO, Land O’Lakes, and TRAIDE Ethiopia, which have not extensively addressed this aspect.

Farmers in the cash crop-based (CCB) dairy production system earn the highest annual income (3983 USD), whereas the other three systems range between 1800 USD to 1995 USD. This increased income in the CCB dairy production system might be due to the higher contribution of income from the sale of cash crops like Khat and coffee in the current study. These results are significantly higher than the reported income for small family farms in Ethiopia (1246 USD) [34], but below the findings of Hussein et al. [35], who reported 4796 USD in Northwest Ethiopia, with 51% of the contribution from Khat. Additionally, the income is much lower compared to Mellisse [36], who found an annual income of 7089 USD for Khat-based farmers in Southern Ethiopia. Other findings also mentioned that Khat can generate income up to fourfold compared to other crops [37].

The CCB system demonstrated a notable trend of higher ownership of improved cattle breeds compared to other systems, likely attributable to the increase in income. This upward trend is further supported by improved access to reproductive technologies such as artificial insemination (AI) and the availability of agro-industrial by-products like concentrates, wheat bran, and noug cake (Tables 2, 3, and 6). Moreover, farmers in the CCB dairy production system benefited in superior dairy cattle housing infrastructure, featuring iron sheet roof and concrete floors (Table 9). Additionally, a subset of these farmers has implemented enhanced manure management practices mainly through the adoption of biogas digester, distinguishing them from their counterparts in other dairy production systems (Fig 3). Consequently, cultivating cash crops emerges as a promising avenue for smallholder farmers seeking to enter the capital-intensive dairy farming sector, particularly in agro-ecological zones conducive to such crops. This assertion is supported by a study conducted in southern Ethiopia, which highlights the significance of income in driving better investment, improved farm management, and fostering agricultural innovation [38].

**Table 9:** Dairy cattle housing across dairy production system.

Description	Dairy production systems								χ2/P-value
	CCB		EB		CB		DCB		
	N = 60		N = 101		N = 87		N = 112		
	F	%	F	%	F	%	F	%	
*Housing*									
Same compound with the family	6	10	46	46	48	55	54	48	33.463/0.00
Separate house	54	90	55	55	39	45	58	52	
*Floor type*									
Bare soil	14	23	49	49	72	83	91	81	156.244/0.00
Concrete	39	65	9	9	8	7	12	11	
Wooden made	7	12	43	43	7	6	9	8	
*Wall and roof type*									
Wooden + Iron sheet	54	90	45	45	64	74	80	71	55.777/0.00
Briks + Iron sheet	4	7	2	1.98	0	0	4	3.6	
Wooden + Plastic cover	1	1.7	2	1.98	0	0	1	0.9	
Wooden + Wooden (Bamboo)	1	1.7	52	51	23	26	27	24	
*Bedding material for the dairy cattle*									
Straw/Grass	23	38	31	31	55	63	77	69	124.353/0.00
Wooden shaving	5	8.3	46	46	8	9.2	9	8.0	
Bare soil	8	13.3	15	25	19	22	17	15	
Concrete	19	32	9	15	5	5.7	9	8.0	
Arranged stone	5	8.3	0	0	0	0	0	0	

χ2 = chi-square value; DCB = Diversified crop based; CB = Cereal based; CCB = Cash crop based; EB = Enset based

In the EB dairy production system, the current study identified *enset* production residues, primarily the leaf laminas, upper parts of the leaf sheaths, and the core of the central shoot, serve as crucial dairy cattle feed resource. This finding supports the observation made by Yilma [10]. The smallholder farmers in this production system are also characterized by having larger farm sizes compared to CB and larger local cattle breeds as well as more allocated grazing land compared to CCB and CB dairy production systems (Table 2). It is also reasonable to allocate more grazing land since they have relatively larger farm sizes and the highest cattle herd sizes per household compared to other DPSs.

These farmers, primarily owning low productivity local cows, rely heavily on indigenous feed resources, including natural pasture, crop residues, and non-conventional feeds such as local beverage by-products (Tables 2 and 3). The increased use of enset production and its residues for cattle feed is attributed to the higher availability, which resulting from extensive enset cultivation and subsequent residue production. This augmented availability is connected to the larger allocation of farmland compared to the other three DPS, as indicated in Table 4. Similarly, the findings by Senbeta et al. [11] indicate that the use of enset as livestock feed is common during both the dry and wet seasons in enset-producing areas, with Heuze et al. [39] reporting that 85% of farmers especially rely on this practice during the dry season. This aligns with the clustering of the Melga district in the Sidama region under the EB dairy production system, as noted by Gizaw et al. [40], who observed enset as the dominant crop and its residues as the main livestock and dairy cattle feed.

Enset integrates well with dairy cattle production, with manure serving as an organic fertilizer for enset and the plant’s residues as cattle feed [41]. Additionally, the EB dairy production system has the highest concentration of barley straw, a key component of cereal crop residues commonly grown in highland agro ecologies (Fig 2A).

Unlike the CCB, the EB system, similar to the DCB, generates lowest annual income (Table 2). This could be attributed to enset being primarily cultivated for household use and the predominance of low productivity indigenous cattle breeds, which results in subsistence oriented rather than market oriented dairy production. Consequently, there is less income generated within the system. Additionally, the enset plant requires a prolonged time to produce the edible products, locally known as *Bula* and Kocho, which generate income at lower prices and slower rates compared to other crops [42]. This slow rate of income generation adversely affects the potential for intensifying dairy cattle productivity.

Smallholder farmers in the CB dairy production system allocated about half of their farmland to cereal crops (Table 2), resulting in more crop residues, especially cereal residues, for dairy cattle feed compared to other systems (Table 4). Additionally, wheat crop residues are more available. This aligns with the work of Hochiso et al. [43] who reported the direct relationship between larger crop land allocation and greater reliance on crop residues for feed. Improved fodder crops, such as desho grass (*Pennisetum pedicellatum*) and phalaris, were more commonly cultivated and used in the CB dairy production system compared to the other three DPSs (Tables 3 and 4). This practice not only enhanced the nutrition and income of the farmers by supporting improved dairy cows but also prevented soil erosion and maintained soil fertility. Some references highlighted the importance of planting forage strips along contours for soil erosion control and conservation [44, 45]. Cultivating improved forages can enhance livestock feed quality by mixing them with cereal crop residues, which in turn improves dairy cattle productivity at smallholder farmers’ level. This approach is supported by reference Mengesha et al. [46], which indicates that combining crop residues with forages improves animal feed quality. Additionally, the DCB dairy production system’s diverse crop cultivation (Tables 2 and 4) helps meet animals nutritional and energy requirements through various crop residues. The DCB system supplies more total root crop residue than the other three DPS, and it is further diversified with other types of crop residues Using diverse crop resides as feed is essential for fulfilling the nutritional requirements of dairy cattle, positively impacting their growth, weight gain, and milk yield [47].

### 4.2 Is a shift to cash crop a promise for intensification of dairy production systems?

A transition from enset, a perennial food crop, to khat (*Catha edulis*) is becoming the new practice among smallholder farmers in the study areas. This shift is driven by the diminishing farmland in response to the increasing population and urbanization. Earlier studies reported expansion of *khat* from 6 to 35% of the area share per farm, which resulted in a decline in the combined area share of *enset* and coffee from 45 to 25% between 1991 and 2013 in Wondo Genet district [21], which is located within the same region as the current study areas. As highlighted earlier, farmers in the CCB DPS allocated over half of their farmland to cash crop mainly *khat* production and characterized by raising more improved dairy cattle under intensive or stall feeding systems supplementing their animals with agro-industrial products (Tables 2, 3 and 5). This implies the direct relationship between intensification of cash crop production and market oriented dairy production in this DPS. In line with the current result, an earlier report indicated the shift from conventional grazing to feeding of industrial by-products as improved dairy cattle breeds are increasingly introduced, accompanied by improved housing as a pathway towards dairy intensification [48]. The proximity to major marketplaces favored the production and marketing of *khat*, a highly perishable cash crop, which enabled farmers to invest, part of the income generated from the sale of lucrative cash crop, on improved cattle breeds, construction of improved housing and purchase agro-industrial by-products to feed their animals (Tables 2, 3, and 9). In agreement with the finding of the current study, Nioroge et al. [49] also indicated the incorporation of concentrate feeds at higher proportion in dairy cattle ration to be a good indicator for the transition towards intensified dairy production.

According to a study, maintaining improved dairy cattle breeds can be considered an intensification strategy for dairy production, serving as a backup revenue source and countermeasure after the collapse of the khat market in the study area [36]. The existence of small grazing lands, which, in the CCB DPS, accounts for about 7% of the farmland pushed farmers to practice intensive stall-feeding method (Tables 1 and 5). Earlier studies reported intensification of dairy feeding systems in response to the diminishing grazing land [50, 51]. Moreover the income gain in CCB DPS might also contribute the smallholder farmers’ advance in some dairy related technologies utilization such as biogas utilization. According to Oenema and Oenema [51] and Clay et al. [52] reported that the dairy operations should depend heavily on external inputs such as waste management, emerging innovations together with off-farm produced supplementary feeds and clean drinking water for the animals. The important use of manure in fertilizing crop lands, particularly in the enset-coffee-based farming system of the peri-urban dairy production system in Dale and Dilla areas, is highlighted in reference [7].

### 4.3 Future sustainable management of smallholder dairy production system

The earlier classification system, based primarily based on agro-ecology, land use, and location at the landscape level, lacked a management strategy specific to dairy production systems. Earlier studies suggested ways forward based on existing conditions to enhance productivity in the future [7, 53]. However, in this study, the classification of the dairy production system was based in the farm-level allocation of different crops and grazing land, highlighting robust crop-livestock integration from the farm to the landscape level. This approach aids in designing tailored management options applicable at the farm level for each production system. Considering the distinctive characteristics of the Cash Crop-Based (CCB) dairy production system, marked by earning of better income from rearing of improved dairy cattle breeds, heightened milk productivity, and situated in a better access to market places, we recommend pursuing a comprehensive strategy. Firstly, further investment in improved dairy cattle breeds is crucial to optimize milk productivity and profitability. Additionally, establishing and strengthening breeding and genetic programs tailored to specific needs maximizes the potential of these breeds. Comprehensive training for farmers on dairy cattle management, nutrition, and healthcare is essential to unlock their full productivity potential. Infrastructure development, including modern milking facilities, cooling systems, and transportation networks, is crucial to maintain milk quality and ensure timely delivery to marketplaces. Facilitating market linkages and supporting the development of value-added products can capture greater value. Lastly, establishing cooperatives can provide easier access to inputs like feed and facilitate product delivery to market, benefiting CCB dairy producers. These measures aim to strengthen the milk and milk products value chain for sustainable and inclusive dairy production. The success of these initiatives requires collaboration between the government, private sector, and non-governmental organizations (NGOs) to ensure sustainable management and enhancement of dairy cattle productivity and marketing of the system.

In the context of the Cereal-Based (CB) dairy production system, where residues primarily come from cereal crops, a strategic measure involves designing a feed treatment specifically tailored for cereal crop residue. Examples of such treatments include urea treatment and the use of Effective Microorganisms (EM) Bokashi, which has been shown to enhance the digestibility of fibrous feeds like crop residues [54, 55]. Additionally, strengthening the existing practice of growing fodder alley cropping with cereal crops will ease dry season feed shortages. Awareness creation on growing of diverse fodder species with different nutritive value would be crucial for future sustainability.

The Enset-Based Dairy Production System (DPS) presents unique challenges due to the utilization of the enset plant as a major food source for humans and feed for livestock. Thus, enhancing enset production and productivity on one hand helps to supply the most nutrient-rich enset leaf to dairy cattle, and on the other hand, contributes to enhancing food self-sufficiency for smallholder farmers. Given that manure is a primary input for enset [42, 56] increasing manure application can yield more enset biomass and residue for feed, thereby enhancing milk productivity. This boost in milk productivity serves to complement the protein-deficient staple food, kocho, extracted from enset [57–59].

Consequently, the improved integration of enset and dairy will strengthen the mutual coexistence of these systems.

In the Diversified Crop-Based Dairy Production System (DPS), elements from both cereal and enset-based systems are integrated. The emphasis lies on cultivating diverse fodder crops alongside cereals and enhancing enset production. This tailored strategy leverages the strengths of both systems to create a more effective and sustainable management plan.

## 5. Conclusion

The study revealed that smallholder farmer in the highlands of southern Ethiopia employ a variety of dairy production systems. Four dairy production systems were distinguished based on allocation of farmland for various major crops that include cash crop based (CCB), *enset* based (EB), cereal based (CB), and diversified crop based (DCB). The highest annual income, improved dairy cattle ownership and use of agro industrial by products as livestock feeds coupled with stall feeding practice were identified as characteristics of CCB dairy production system. Furthermore, better manure management has been observed in the system by applying a biogas technology, which requires high investment. The majority of the results from the CCB dairy production system confirm that the involvement in the production of cash crops is what causes the intensification of dairy cattle. As a consequence, it was found that in the CCB, there was a direct correlation between the commercialization of crops and the intensive dairy production system.

In the EB dairy production system, *enset* leftovers are the most readily available feed source for dairy cow producers. These smallholder farmers tend to have larger farms and more grazing land, possess larger herds of local breeds, and generally have minimal experience with cross-breeding. Moreover, a more significant ownership of the herd in EP DPS may be linked to an extensive *enset* plant manure requirement. In the CB DPS, cereal crop leftovers are one of the main feed sources along with an established good tradition of planting improved forage plants that are beneficial for both preventing soil erosion and serving as a feed source for dairy cattle. The DCB DPS also had access to and used a range of crop leftovers from the food crop varieties that were grown there. Thus, being aware of each type of DPS and its characteristics will help development in designing policies to enhance dairy productivity among smallholder farmers in Southern Ethiopia. This study suggests promoting biogas technology in CCB systems through financial support and encouraging cash crop production to drive dairy intensification. Enhancing feed resources by cultivating enset and cereals, along with improved crop leftover management, is vital for EB and CB systems. Introducing cross-breeding programs in EB systems can boost herd potential, while supporting forage plant cultivation and crop diversification in CB and DCB systems can improve resilience and productivity. Tailored policies addressing these needs will foster sustainable improvements in dairy farming.

## Supporting information

S1 Data (XLSX)
